# Deep Learning Radiomics Model Based on Computed Tomography Image for Predicting the Classification of Osteoporotic Vertebral Fractures: Algorithm Development and Validation

**DOI:** 10.2196/75665

**Published:** 2025-08-29

**Authors:** Jiayi Liu, Lincen Zhang, Yousheng Yuan, Jun Tang, Yongkang Liu, Liang Xia, Jun Zhang

**Affiliations:** 1 Department of Radiology, Sir Run Run Hospital, Nanjing Medical University, 109 Longmian Road, Nanjing, 211100, China, 86 18851667275; 2 Department of Radiology, The Affiliated Taizhou People's Hospital of Nanjing Medical University, Taizhou, China; 3 Department of Radiology, The Affiliated Hospital of Nanjing University of Chinese Medicine, Nanjing, China

**Keywords:** deep learning, radiomics, osteoporotic vertebral fractures, tomography, classification, model interpretability, x-ray computed

## Abstract

**Background:**

Osteoporotic vertebral fractures (OVFs) are common in older adults and often lead to disability if not properly diagnosed and classified. With the increased use of computed tomography (CT) imaging and the development of radiomics and deep learning technologies, there is potential to improve the classification accuracy of OVFs.

**Objective:**

This study aims to evaluate the efficacy of a deep learning radiomics model, derived from CT imaging, in accurately classifying OVFs.

**Methods:**

The study analyzed 981 patients (aged 50‐95 years; 687 women, 294 men), involving 1098 vertebrae, from 3 medical centers who underwent both CT and magnetic resonance imaging examinations. The Assessment System of Thoracolumbar Osteoporotic Fractures (ASTLOF) classified OVFs into Classes 0, 1, and 2. The data were categorized into 4 cohorts: training (n=750), internal validation (n=187), external validation (n=110), and prospective validation (n=51). Deep transfer learning used the ResNet-50 architecture, pretrained on RadImageNet and ImageNet, to extract imaging features. Deep transfer learning–based features were combined with radiomics features and refined using Least Absolute Shrinkage and Selection Operator (LASSO) regression. The performance of 8 machine learning classifiers for OVF classification was assessed using receiver operating characteristic metrics and the “One-vs-Rest” approach. Performance comparisons between RadImageNet- and ImageNet-based models were performed using the DeLong test. Shapley Additive Explanations (SHAP) analysis was used to interpret feature importance and the predictive rationale of the optimal fusion model.

**Results:**

Feature selection and fusion yielded 33 and 54 fused features for the RadImageNet- and ImageNet-based models, respectively, following pretraining on the training set. The best-performing machine learning algorithms for these 2 deep learning radiomics models were the multilayer perceptron and Light Gradient Boosting Machine (LightGBM). The macro-average area under the curve (AUC) values for the fused models based on RadImageNet and ImageNet were 0.934 and 0.996, respectively, with DeLong test showing no statistically significant difference (*P*=2.34). The RadImageNet-based model significantly surpassed the ImageNet-based model across internal, external, and prospective validation sets, with macro-average AUCs of 0.837 versus 0.648, 0.773 versus 0.633, and 0.852 versus 0.648, respectively (*P*<.05). Using the binary “One-vs-Rest” approach, the RadImageNet-based fused model achieved superior predictive performance for Class 2 (AUC=0.907, 95% CI 0.805‐0.999), with Classes 0 and 1 following (AUC/accuracy=0.829/0.803 and 0.794/0.768, respectively). SHAP analysis provided a visualization of feature importance in the RadImageNet-based fused model, highlighting the top 3 most influential features: cluster shade, mean, and large area low gray level emphasis, and their respective impacts on predictions.

**Conclusions:**

The RadImageNet-based fused model using CT imaging data exhibited superior predictive performance compared to the ImageNet-based model, demonstrating significant utility in OVF classification and aiding clinical decision-making for treatment planning. Among the 3 classes, the model performed best in identifying Class 2, followed by Class 0 and Class 1.

## Introduction

Osteoporotic vertebral fractures (OVFs) have a subtle onset and complex progression, affecting about 40% of postmenopausal women and 25%‐33% of older men. In China, a new OVF case is reported approximately every 17.4 seconds [1]. OVFs are linked to high disability and mortality rates [2]. Accurate classification of OVFs is widely recognized as critical for early diagnosis, treatment planning, and prognosis evaluation [3]. Current classification systems, including the Genant semi-quantitative method [4], Heini classification [5], osteoporotic fractures classification [6], and the Assessment System of Thoracolumbar Osteoporotic Fractures (ASTLOF) [7], differ in methodology but lack global agreement [8]. Among these, the ASTLOF system has demonstrated good reproducibility and clinical utility, integrating vertebral morphology, magnetic resonance imaging (MRI) signal characteristics, bone mineral density (BMD), and pain severity into a preoperative scoring framework. This system supports targeted treatment selection and provides valuable clinical guidance [9]. Accordingly, the ASTLOF classification was adopted as the standard in this study.

Computed tomography (CT) imaging, with its high spatial resolution, allows for detailed observation of subtle changes in vertebral endplates, cortical bone, and cancellous bone, providing a more reliable basis for OVF classification and clinical guidance [10]. Currently, CT equipment is widely available in secondary and tertiary hospitals, and some community hospitals have also introduced CT systems [11]. CT imaging is crucial for accurately classifying OVFs and has substantial clinical importance. Radiomics aids in analyzing trabecular bone microstructure [12], assessing BMD [13], differentiating acute from chronic OVFs [14], and predicting residual back pain in these patients [15]. Deep learning radiomics (DLR) uses network architectures such as ResNet, pretrained on ImageNet, to extract deep imaging features from images, a widely adopted approach. RadImageNet, as an open-access, public medical imaging dataset, theoretically provides better performance for deep transfer learning (DTL) in medical imaging tasks compared to ImageNet [16]. Our research team has preliminarily validated this hypothesis [17]. However, whether it can improve the performance of 3-class prediction models still requires further exploration and verification.

In this study, we used thoracolumbar medical imaging data from multiple health care centers. We applied DTL on both RadImageNet and ImageNet datasets to extract DTL features and also used the open-source PyRadiomics package (developed by the Computational Imaging & Bioinformatics Lab, Brigham and Women’s Hospital/Harvard Medical School; lead developer: Joost J. M. van Griethuysen) to extract traditional radiomics features. We developed a predictive model for OVF classification using CT imaging by integrating and selecting DTL and radiomics features within the ASTLOF system. The model was validated, tested, and compared for its predictive performance using multicenter data.

## Methods

### Patient Selection

This study used medical imaging data from 3 Chinese hospitals, with ethics committee approval from each institution. The retrospective study design negated the need for informed patient consent. CT and MRI data from patients diagnosed with OVFs at Center I (Taizhou People’s Hospital, Nanjing Medical University) and Center II (Affiliated Hospital of Nanjing University of Chinese Medicine) between December 2018 and December 2024 were used to create the training, internal validation, and external validation datasets. Textbox 1 shows the inclusion criteria and exclusion criteria. An independent test dataset was acquired from Center III (Sir Run Run Hospital, Nanjing Medical University) spanning January to December 2024. The dataset’s inclusion and exclusion criteria matched those of the training and validation datasets.

**Figure 1.**
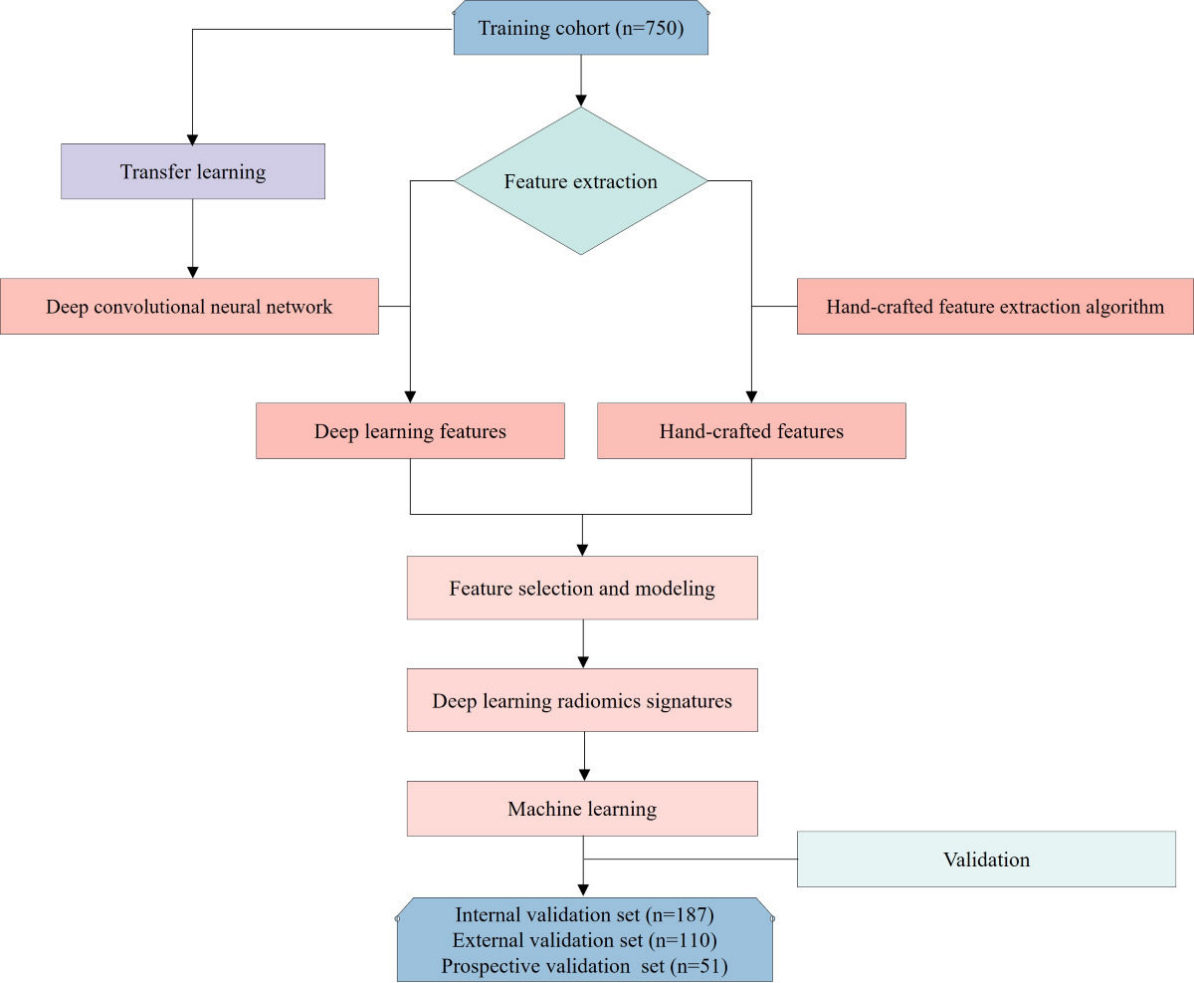
The flowchart in this study outlines the key steps and processes involved in the research workflow.

Figures1  2 provide detailed information on case collection, grouping, image preprocessing, feature extraction, analysis, and model development through flowcharts and the DLR workflow.

Textbox 1. **Inclusion criteria**

- Patients aged ≥50 years, meeting osteoporotic vertebral fracture diagnostic criteria [18], with no trauma history or only minor trauma.
- Complete computed tomography and magnetic resonance imaging DICOM data, with no more than a 2-week interval between the 2 scans.
- Comprehensive clinical records encompassing gender, age, and dual-energy X-ray absorptiometry outcomes.
- Clinical presentations, such as absence of significant pain, back pain triggered by posture, persistent pain, or neurological symptoms.

**Exclusion criteria**

- Suspected infections or pathological fractures related to tumors.
- Poor image quality or artifacts caused by foreign objects.
- Uncertain osteoporotic vertebral fracture classification.

**Figure 2.**
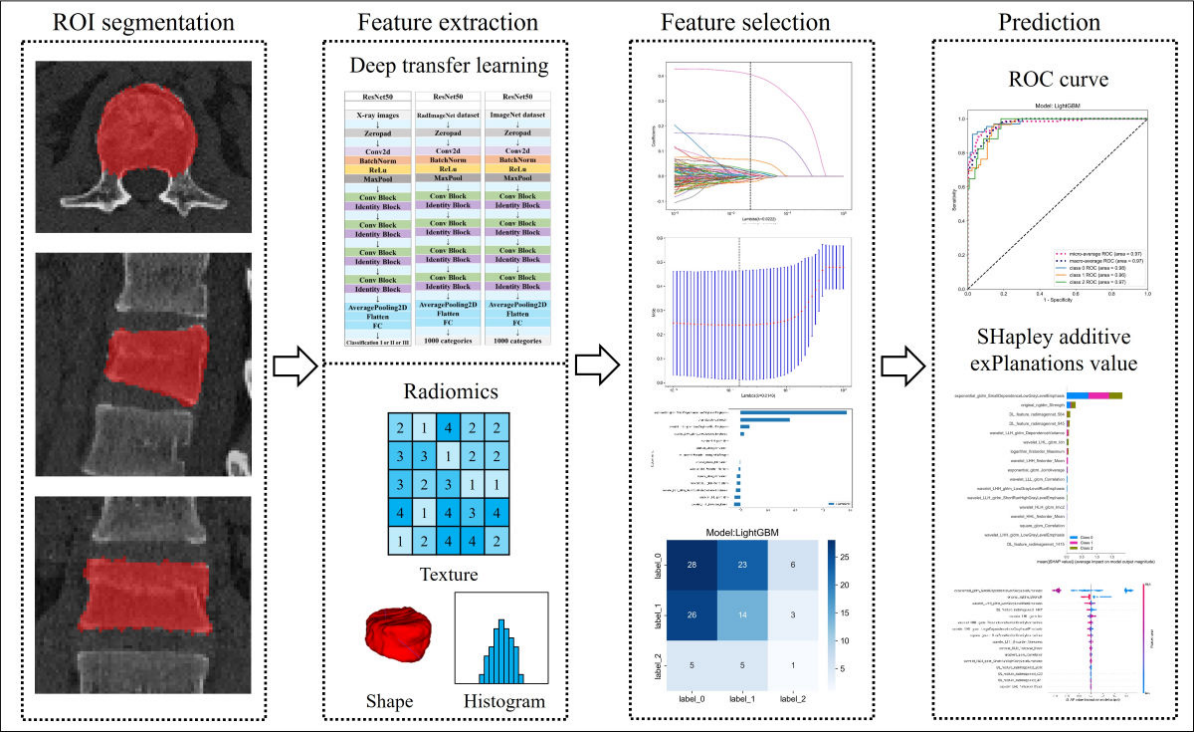
The workflow of the deep learning radiomics process illustrates the systematic steps involved in data processing, feature extraction, model development, and validation. ROC: receiver operating characteristic; ROI: region of interest.

### CT and MRI Acquisition Protocol

#### CT Scans

CT scans were performed across 3 centers using multidetector or dual-source CT systems, including: GE Lightspeed Ultra (16-slice, USA), Siemens Somatom Definition (128-slice and 256-slice, Germany), Siemens Sensation64 (64-slice, Germany), Philips Brilliance iCT (256-slice, Netherlands), GE Optima CT670 (64-slice, USA). Key scan parameters were: tube voltage 120 kVp (with or without automated current modulation), tube current 118‐320 mA (with or without automated current modulation), and image matrix 512×512, and layer thickness and interval 1 mm.

#### MRI Scans

MRI examinations were conducted on 3.0T scanners from Siemens (Verio, Skyra, and Prisma), Philips (Ingenia CX and Achieva TX), across the 3 centers. Image sequences included short tau inversion recovery (STIR) and T2-weighted fat-suppressed images. All patients underwent STIR or T2-weighted fat-suppressed MRI scans. Additional details regarding the imaging devices and parameters for both CT and MRI are available in Multimedia Appendix 1.

### Classification

The ASTLOF system classifies OVFs by assigning scores based on vertebral morphology, MRI findings, BMD, and clinical symptoms. Changes in morphology seen in CT or MRI scans are rated as normal (0 points), compression (1 point), or burst fracture (2 points). MRI assessments use sagittal T2-weighted fat-suppressed or STIR sequences, assigning scores based on normal appearance (0 points), high signal alterations (1 point), or the presence of vacuum or fluid signs within vertebrae (2 points). BMD is assessed via T-scores, with values >−2.5 scoring 0, between −2.5 and −3.5 scoring 1, and ≤−3.5 scoring 2. Clinical symptoms are categorized as no significant pain (0 points), positional low back pain (1 point), or persistent pain or neurological symptoms (2 points). No significant pain refers to an absence of discomfort during daily activities, while positional low back pain is triggered by specific postures such as prolonged standing, sitting, or bending. Persistent pain is continuous and unrelieved by rest or posture changes, while neurological symptoms indicate nerve involvement, manifesting as numbness, tingling, or muscle weakness in the lower limbs. OVFs are classified based on total scores: Class 0 (≤3) for conservative treatment, Class 1 (=4) for either conservative or surgical treatment, and Class 2 (≥5) for surgical intervention. Evaluation scores were independently determined by 2 musculoskeletal radiologists (Doctor A with 6 years of experience and Doctor B with 10 years of experience), with disagreements resolved through discussion and consensus.

### Clinical and CT Images Evaluation

Patient information, including age, gender, dual-energy X-ray absorptiometry (DXA)-measured T-scores, and treatment details, was obtained from the clinical case management system. CT images were obtained using a bone window setting (width 1500 and level 500) and reconstructed with a 1-mm slice thickness for subsequent processing and analysis.

### Region of Interest Segmentation

**Figure 3.**
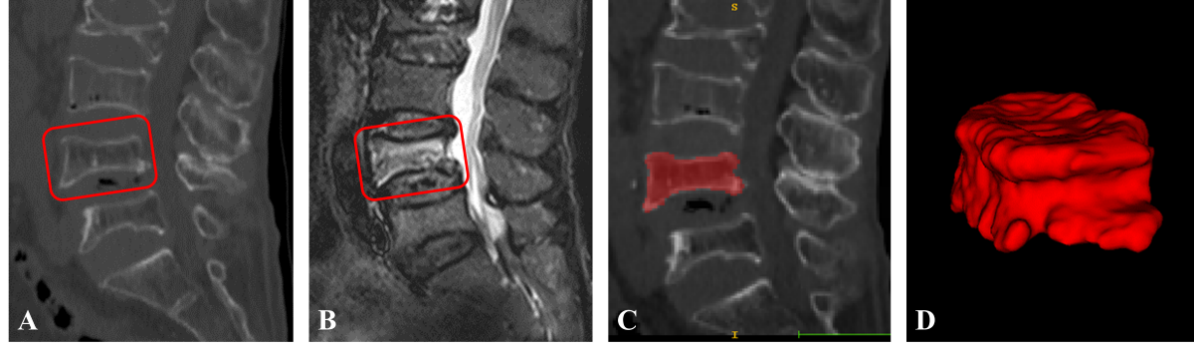
Segmentation of a fractured vertebral body for radiomic analysis in an 82-year-old woman with an acute osteoporotic vertebral fracture is illustrated.(**A**) Sagittal non–contrast-enhanced spine computed tomography images show an osteoporotic vertebral fracture of L4. (**B**) Sagittal T2-weighted fat-suppressed imaging reveals hyperintensity associated with the acute vertebral fracture. (**C**) The region of interest is delineated on sagittal computed tomography images. (**D**) Three-dimensional volume meshes are reconstructed to visualize the segmentation.

Radiologists manually segmented fractured vertebrae using ITK-SNAP software (version 3.8.0; developed by the Penn Image Computing and Science Laboratory, University of Pennsylvania; lead developer: Paul A. Yushkevich) in this study. Radiologist A outlined and filled the edges of the fractured vertebrae on the CT images to create regions of interest, carefully excluding adjacent intervertebral discs, pedicles, and surrounding tissues to ensure precise delineation. The segmented masks were then saved as “nii” files for further analysis (Figure 3). Interobserver agreement was evaluated by having Radiologist A and Radiologist B independently resegment a random subset of 30 patients from the training dataset after 1 month, using the intraclass correlation coefficient (ICC) for assessment.

### Radiomics and DTL Features Extraction

All images were resampled using B-spline interpolation and standardized with Z-score normalization to reduce variability across centers. Feature extraction algorithms were standardized in accordance with the Image Biomarker Standardization Initiative [19]. Radiomic features, encompassing first-order, shape, and texture characteristics, were extracted using the open-source Python package PyRadiomics (developed by the Computational Imaging & Bioinformatics Lab, Brigham and Women’s Hospital/Harvard Medical School; lead developer: Joost J. M. van Griethuysen) [20]. These texture features include the gray level co-occurrence matrix, gray level size zone matrix, gray level run length matrix, neighboring gray tone difference matrix, and gray level dependence matrix. For comprehensive details on the extracted features, refer to the PyRadiomics documentation [21]. To minimize variations across centers, the Combat method was applied for feature harmonization [22]. To mitigate bias and minimize overfitting risks from excessive features, a 2-step feature selection process was implemented: initially, features demonstrating strong reliability were retained using ICC evaluation, followed by further selection through the Least Absolute Shrinkage and Selection Operator (LASSO) algorithm.

Transfer learning is used because retraining a convolutional neural network for a specific task demands extensive image data and intricate parameter configurations, which are challenging to obtain in this study. Transfer learning involves fine-tuning a pretrained deep learning network to adapt it for a new task, allowing deep learning to be applied effectively on smaller datasets. Images were resampled to 64×64 (as is common practice in deep learning pipelines) and pixel intensities normalized to a mean of 0 and SD of 1. We acknowledge that resampling to 64×64 may lead to some loss of spatial detail. However, we chose this size after preliminary experiments demonstrated that it retained sufficient image features for accurate classification, while balancing computational efficiency and memory requirements. The DTL approach, akin to previous studies [23], was implemented using the Python 3.6-based deep learning library (Guido van Rossum), PyTorch. The study used ResNet50 as the foundational model (MultimediaAppendices 2  3).

To execute transfer learning effectively, the learning rate was carefully configured. Features were extracted from the model’s penultimate layer (AveragePooling), with model parameters divided into backbone and task-specific components. The backbone component used pretrained parameters from RadImageNet [24] or ImageNet for initialization, whereas the task-specific component was initialized randomly. Drawing inspiration from the cosine annealing learning rate decay algorithm, optimizations were implemented by fine-tuning the backbone component with pretrained weights only when essential to maintain transfer learning quality. Concurrently, task-specific parameters were modified according to task demands, enabling the model to effectively adapt to the target data.

### Data Dimensionality Reduction

To identify reproducible and nonredundant radiomic features, a systematic process was implemented. First, features with ICC ≥0.8 from 2 independent evaluations were retained for reproducibility [25]. Redundancy was minimized by computing the Spearman rank correlation coefficient between features. Features with a correlation above 0.9 were subjected to a greedy recursive elimination strategy to remove the most redundant ones, ensuring overall representation was maintained. Stable features were then selected using the LASSO algorithm, which applies a penalty parameter (λ) to shrink regression coefficients, retaining only relevant features. The optimal λ value was determined through 10-fold cross-validation, and features with nonzero coefficients were selected for the final set. To further reduce redundancy, correlated features with a coefficient greater than 0.5 were excluded, resulting in a refined subset of independent features. For DTL features (initially with a dimension of 2048), principal component analysis was applied to reduce dimensionality, balancing deep learning and radiomic features while mitigating overfitting. The chosen radiomic and deep learning features were combined through early fusion to create a unified feature set, with all features standardized using Z-score normalization for compatibility. Finally, after fusion, LASSO-Cox regression was applied to select the most robust features, which were further refined through dimensionality reduction to define an optimal subset. This carefully curated feature set represented the most relevant combination of radiomic and deep learning features, facilitating reliable model development.

### Model Development

To prevent data leakage, all features used for building the predictive model were exclusively derived from the training set. Machine learning models were implemented using the scikit-learn library following feature selection and fusion. The models comprised logistic regression (LR), support vector machine (SVM), k-nearest neighbor (KNN), decision tree (DT), random forest (RF), extremely randomized trees (ExtraTrees), eXtreme gradient boosting (XGBoost), Light Gradient Boosting Machine (LightGBM), and multilayer perceptron (MLP). We observed an imbalance in the distribution of samples among the ASTLOF classification categories, with notably fewer cases in Class 2. To reduce the risk of biased model performance, we apply strategies such as class weighting during model training. Model training was conducted using the training set and optimized through grid search with adjustable parameters specific to each algorithm. Model performance was assessed using 5-fold cross-validation on the training data, selecting the best parameters to construct the optimal fused-feature model. Using a larger k (such as 10 or more) would have increased computational cost and training time substantially, without necessarily providing a significant improvement in model assessment, especially given the size of our dataset. Therefore, 5-fold cross-validation was appropriate for our study while maintaining a reasonable balance between thoroughness and practicality. The receiver operating characteristic (ROC) curve was plotted, and model accuracy was validated through 1000 iterations of bootstrap resampling. Performance metrics such as area under the curve (AUC), accuracy (ACC), sensitivity (SEN), and specificity (SPE) were evaluated. Finally, statistically significant clinical baseline characteristics were integrated with the best fused feature model to develop a combined model, which was visualized through a nomogram.

This study used the “One-vs-Rest” (OvR) strategy for multiclass tasks by decomposing the problem into several binary classification tasks. When Class 0 was labeled positive, Classes 1 and 2 were negative; similarly, labeling Class 1 or 2 as positive made the others negative, creating 3 OvR classification models. Model performance was assessed by plotting ROC curves and calculating metrics including AUC, ACC, SEN, and SPE. The generalization ability was evaluated using internal, external validation, and test datasets. Macro- and micro-average AUC were used for a thorough assessment of multiclass tasks [26]. Macro-average AUC computes the AUC for each class and averages them equally, which can be less representative in cases of significant class imbalance. Conversely, micro-average AUC aggregates predictions from all classes into a single confusion matrix, emphasizing the influence of larger sample sizes and providing a better reflection of overall performance on imbalanced datasets.

### Data Analysis

Statistical analyses were conducted using R software (R Core Team; version 4.0.3), and radiomics and deep learning models were developed and implemented on Python 3.7 (Python Software Foundation). Continuous variables are presented as mean (SD), while categorical variables are shown as counts or percentages. Independent samples *t* tests were used to assess differences in continuous variables, while chi-square tests were applied for comparisons of categorical variables. The DeLong test was used to compare ROC curves and assess the predictive models’ overall performance. In addition, bootstrap validation with 1000 resamples was conducted to ensure robust evaluation of model accuracy. *P*<.05 was considered statistically significant, serving as a benchmark for assessing the reliability of observed differences and associations. These comprehensive statistical methods ensured the rigor of model evaluation and the clarity of results interpretation.

### Explainable Artificial Intelligence

The Shapley Additive Explanations (SHAP) method (GitHub, Inc [27]) was used to assess the importance of various features by calculating their contributions to prediction outcomes, offering a clear explanation of their significance [28]. Using SHAP values, the predictive output for each sample is decomposed into individual feature contributions, providing a quantifiable measure of feature influence. The magnitude of a SHAP value indicates the extent of a feature’s influence on the model’s prediction, where positive values signify a positive impact and negative values signify a negative impact. For example, in a disease prediction model, a feature with a SHAP value greater than 0 suggests it increases the predicted likelihood of disease occurrence, whereas a value below 0 implies a reduced likelihood. Beyond individual predictions, SHAP also ranks features by their overall importance across the model and reveals the relationships between features and prediction outcomes. This integration of quantitative contribution, directional influence, and feature importance ranking facilitates a comprehensive understanding of the model’s decision-making, revealing how particular features influence predictions and their significance.

### Ethical Considerations

This study comprised a retrospective component and a prospective validation cohort. For the retrospective component, the local Ethics Committees of the Affiliated Hospital of Nanjing University of Chinese Medicine and the Affiliated Taizhou People’s Hospital of Nanjing Medical University waived the requirement for ethical approval and informed consent because the analysis involved existing data collected during routine clinical care and posed minimal risk to participants. The prospective validation cohort was approved by the Institutional Ethics Committee of Sir Run Run Hospital, Nanjing Medical University, on November 25, 2023 (approval no. 2023-SR-055). All participants in the prospective cohort provided written informed consent before enrollment. To protect privacy, all images and relevant data were deidentified prior to analysis and reporting. No individually identifiable information was used. Participants did not receive financial or other material compensation for participation. The study was conducted in accordance with the principles of the Declaration of Helsinki and relevant institutional guidelines and regulations.

## Results

### Clinical Features of the Studied Patients

**Figure 4.**
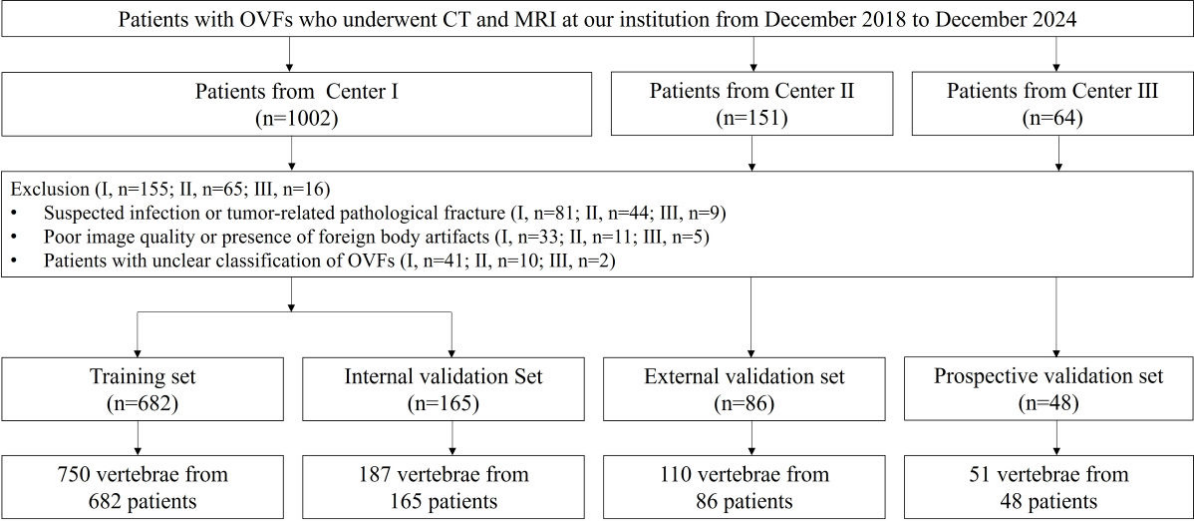
The flowchart summarizes patient selection and allocation to the training set, internal and external validation set, and prospective validation set of the multicenter study. CT: computed tomography; MRI: magnetic resonance imaging; OVFs: osteoporotic vertebral fractures.

The study enrolled 981 patients aged 50 to 95 years, with an average age of 69.56 (9.88) years. Of these, 687 were females (70%) and 294 were males (30%). Based on T-scores, 30 patients (3.1%) were classified as having normal bone mass, 257 (26.2%) as having low bone mass, and 694 (70.7%) as having osteoporosis. Among the participants, 87 patients presented with 2 OVFs, and 15 patients had 3 OVFs, resulting in a total of 1098 fractured vertebrae included in the analysis. The dataset was partitioned into a training set (750 cases, 68.4%), an internal validation set (187 cases, 17%), an external validation set (110 cases, 10%), and a prospective validation set (51 cases, 4.6%). Table 1 summarizes the demographic and clinical characteristics of each dataset, and Table 2 details the treatment conditions across the 3 classifications. Figure 4 illustrates the case selection process, emphasizing the random 8:2 allocation of cases into the training and internal validation sets.

**Table 1.** Baseline characteristics of patients with osteoporotic vertebral fracture in the training, internal and external validation, and prospective validation cohorts.

Characteristics	Training set (n=750)	Interval validation set (n=187)	External validation set (n=110)	Prospective validation set (n=51)
Sex, n (%)				
Female	541 (72.1)	138 (73.8)	78 (70.9)	36 (70.6)
Male	209 (27.9)	49 (26.2)	32 (29.1)	15 (29.4)
Age (years)				
Mean (SD)	68.25 (11.18)	70.19 (10.56)	69.56 (10.23)	69.51 (10.32)
DXA^a^ T-score				
Mean (SD)	−2.82 (0.82)	−2.79 (0.81)	−2.85 (0.75)	−2.83 (0.77)
Fracture location, n (%)				
Thoracic	247 (32.9)	52 (27.8)	32 (29.1)	17 (33.3)
Lumbar	503 (67.1)	135 (72.2)	78 (70.9)	34 (66.7)
Fracture staging, n (%)				
Acute	492 (65.6)	112 (59.9)	69 (62.7)	33 (64.7)
Chronic	258 (34.4)	75 (40.1)	41 (37.3)	18 (35.3)
ASTLOF^b^ score, n (%)				
1‐3 points	345 (46.0)	88 (47.1)	51 (46.4)	24 (47.1)
4 points	338 (45.1)	76 (40.6)	46 (41.8)	22 (43.1)
5‐8 points	67 (8.9)	23 (12.3)	13 (11.8)	5 (9.8)
Therapeutic method, n (%)				
Conservative treatment	435 (58)	106 (56.7)	62 (56.3)	28 (54.9)
PVA^c^	258 (34.4)	73 (39)	40 (36.4)	19 (37.3)
Open surgery	57 (7.6)	8 (4.3)	8 (7.3)	4 (7.8)

a DXA: dual-energy X-ray absorptiometry.

b ASTLOF: Assessment System of Thoracolumbar Osteoporotic Fracture.

c PVA: percutaneous vertebral augmentation.

**Table 2.** Distribution of osteoporotic vertebral fractures based on the Assessment System of Thoracolumbar Osteoporotic Fractures classification and their association with different therapeutic methods.

Classification	Conservative treatment (n=631, %)	PVA^a^ (n=390, %)	Open surgery (n=77, %)
Class 0 (1-3 points)	411 (80.9)	71 (14)	26 (5.1)
Class 1 (4 points)	162 (33.6)	289 (60)	31 (6.4)
Class 2 (5-8 points)	58 (53.7)	30 (27.8)	20 (18.5)

a PVA, percutaneous vertebral augmentation.

### Radiomics Feature Selection (RadImageNet-Based)

The LASSO-Cox regression analysis model was used to perform dimensionality reduction on the fused features. The penalty coefficient (λ=0.0031) was chosen to optimize feature selection, with Multimedia Appendix 4 depicting the changes in feature coefficients as λ varied. Following the final feature selection, 17 radiomics features and 16 DTL features were retained. The DTL_Radscore was constructed using the fused features and their regression coefficients, as shown in Multimedia Appendix 5.

### Radiomics Feature Selection (ImageNet-Based)

The LASSO-Cox regression model, with a penalty coefficient of λ=0.0295, was used to optimally select features by reducing the dimensionality of the fused dataset. Multimedia Appendix 6 displays the feature selection process and the curve showing the change in feature coefficients with λ. Following the final selection, 17 radiomics features and 37 DTL features were retained. Using these fused features and their associated regression coefficients, the DTL_Radscore was constructed, as detailed in Multimedia Appendix 7.

### Overall Validation of Different Radiomics Models

**Figure 5.**
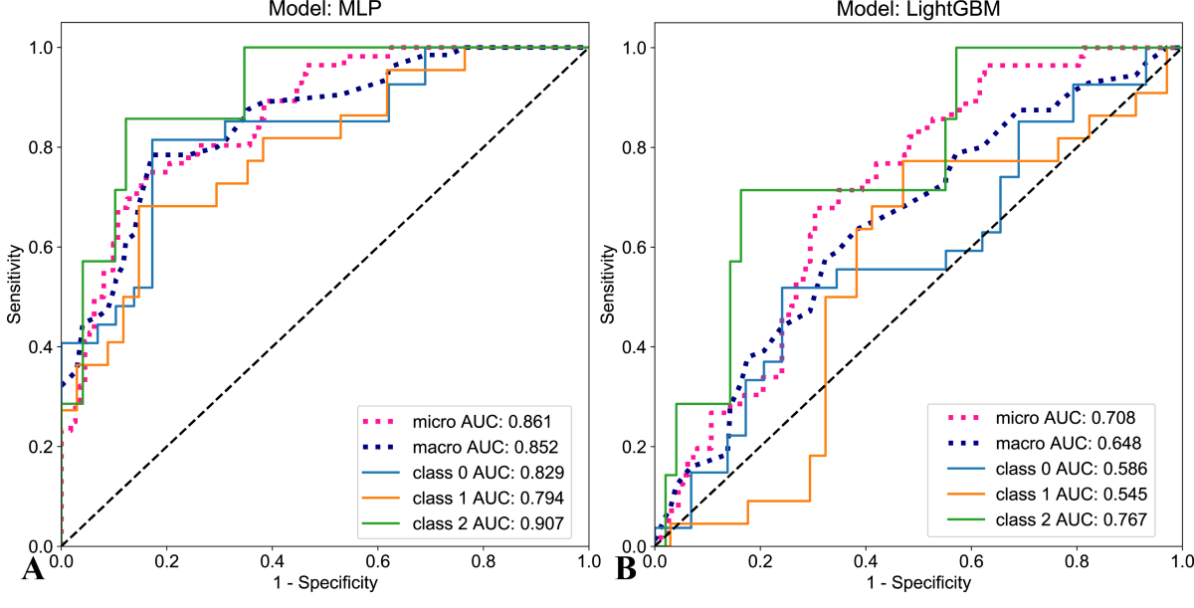
The receiver operating characteristic curves for the predictive performance of the 2 models (A. RadImageNet, B. ImageNet). AUC: area under the curve; MLP: multilayer perceptron; LightGBM: Light Gradient Boosting Machine.

**Figure 6.**
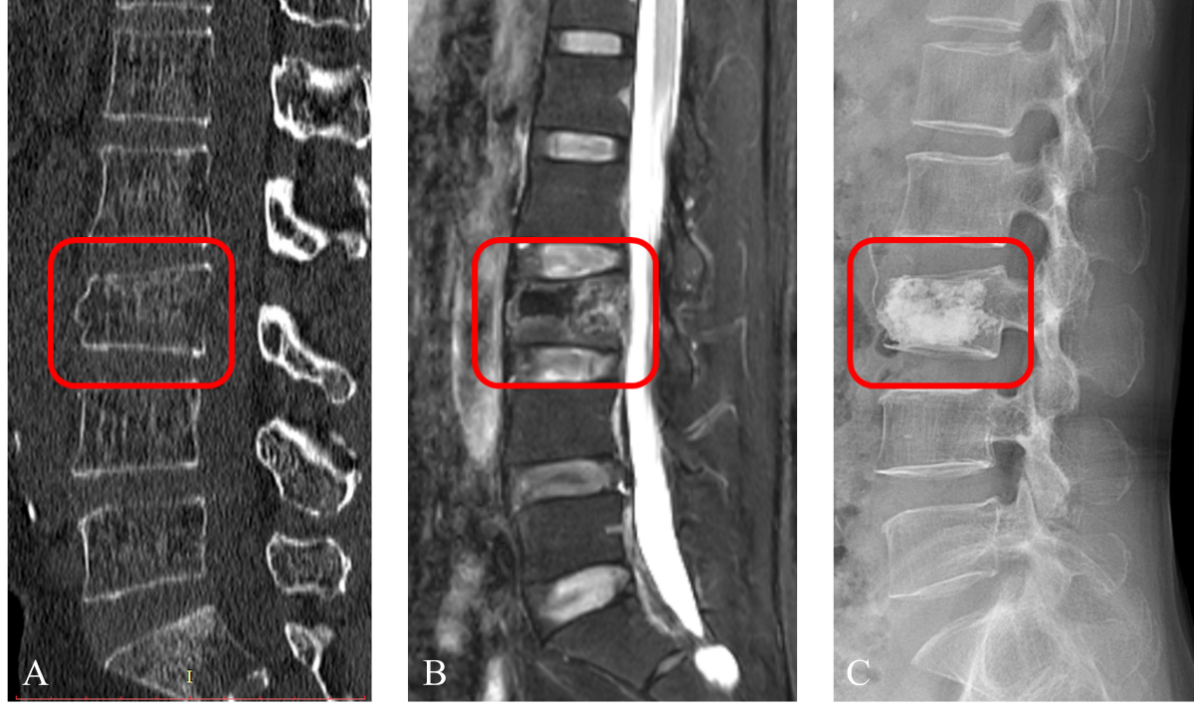
A case from the prospective validation cohort involves a 60-year-old female patient with osteoporotic vertebral fractures and an Assessment System of Thoracolumbar Osteoporotic Fractures score of 6. It was misclassified by the ImageNet model, but was correctly classified by the RadImageNet model. (**A**) Computed tomography imaging; (**B**) MRI imaging; (**C**) Postpercutaneous vertebroplasty showing bone cement leakage.

The optimal machine learning algorithms for fused feature models trained on RadImageNet and ImageNet datasets, based on macro-average AUC, ACC, and *F*_1_-score, were identified as MLP and LightGBM, respectively. Table 3 summarizes the validation results for the 2 fused feature models in the 3-class classification task. In the training set, the DeLong test indicated no statistically significant difference between the 2 fused feature models (0.934 vs 0.996, *P*=2.34). In the internal, external, and prospective validation sets, the RadImageNet-based fused feature model demonstrated significantly higher macro-average AUC values than the ImageNet-based model (0.837 vs 0.648, 0.773 vs 0.633, and 0.852 vs 0.648, respectively), as confirmed by the DeLong test (*P*<.05). Figure 5 displays the ROC curves for both models predicting OVF classifications in the prospective validation set. The RadImageNet-based fused feature model, using the binary OvR strategy, excelled in predicting classification 2 with an AUC of 0.907 and an ACC of 0.857. For classifications 0 and 1, the model achieved AUCs and ACCs of 0.829, 0.803 and 0.794, 0.768, respectively. Figure 6 highlights instances where the ImageNet-based fused feature model made incorrect predictions, while the RadImageNet-based model successfully identified the correct classifications.

**Table 3.** The performance of the models across the training set, internal and external validation sets, and the prospective validation set.

Model	Training set		Interval validation set		External validation set		Prospective validation set	
	Accuracy	AUC^a^	Accuracy	AUC^a^	Accuracy	AUC^a^	Accuracy	AUC^a^
RadImageNet-based								
Class 0	0.867	0.939 (0.924‐0.955)	0.777	0.834 (0.779‐0.889)	0.715	0.794 (0.714‐0.875)	0.803	0.829 (0.719‐0.938)
Class 1	0.825	0.905 (0.884‐0.925)	0.726	0.768 (0.700‐0.836)	0.681	0.747 (0.658‐0.836)	0.768	0.794 (0.673‐0.915)
Class 2	0.886	0.953 (0.934‐0.973)	0.746	0.898 (0.839‐0.957)	0.767	0.756 (0.593‐0.920)	0.857	0.907 (0.805‐0.999)
Three classifications^b^	0.793	0.934 (0.914‐0.951)	0.660	0.837 (0.773‐0.894)	0.647	0.773 (0.655‐0.877)	0.732	0.852 (0.732‐0.951)
ImageNet-based								
Class 0	0.969	0.995 (0.992‐0.997)	0.619	0.619 (0.540‐0.698)	0.655	0.675 (0.576‐0.774)	0.625	0.586 (0.433‐0.739)
Class 1	0.964	0.996 (0.994‐0.999)	0.624	0.576 (0.488‐0.664)	0.560	0.551 (0.445‐0.656)	0.607	0.545 (0.385‐0.705)
Class 2	0.952	0.995 (0.993‐0.999)	0.756	0.737 (0.631‐0.843)	0.621	0.654 (0.480‐0.827)	0.803	0.767 (0.580‐0.953)
Three classifications^b^	0.916	0.996 (0.993‐0.998)	0.533	0.648 (0.553‐0.735)	0.551	0.633 (0.501‐0.753)	0.429	0.648 (0.466‐0.799)

a Data in parentheses are 95% CIs.

b Date are macro-average.

### Feature Contribution and Model Interpretation

**Figure 7.**
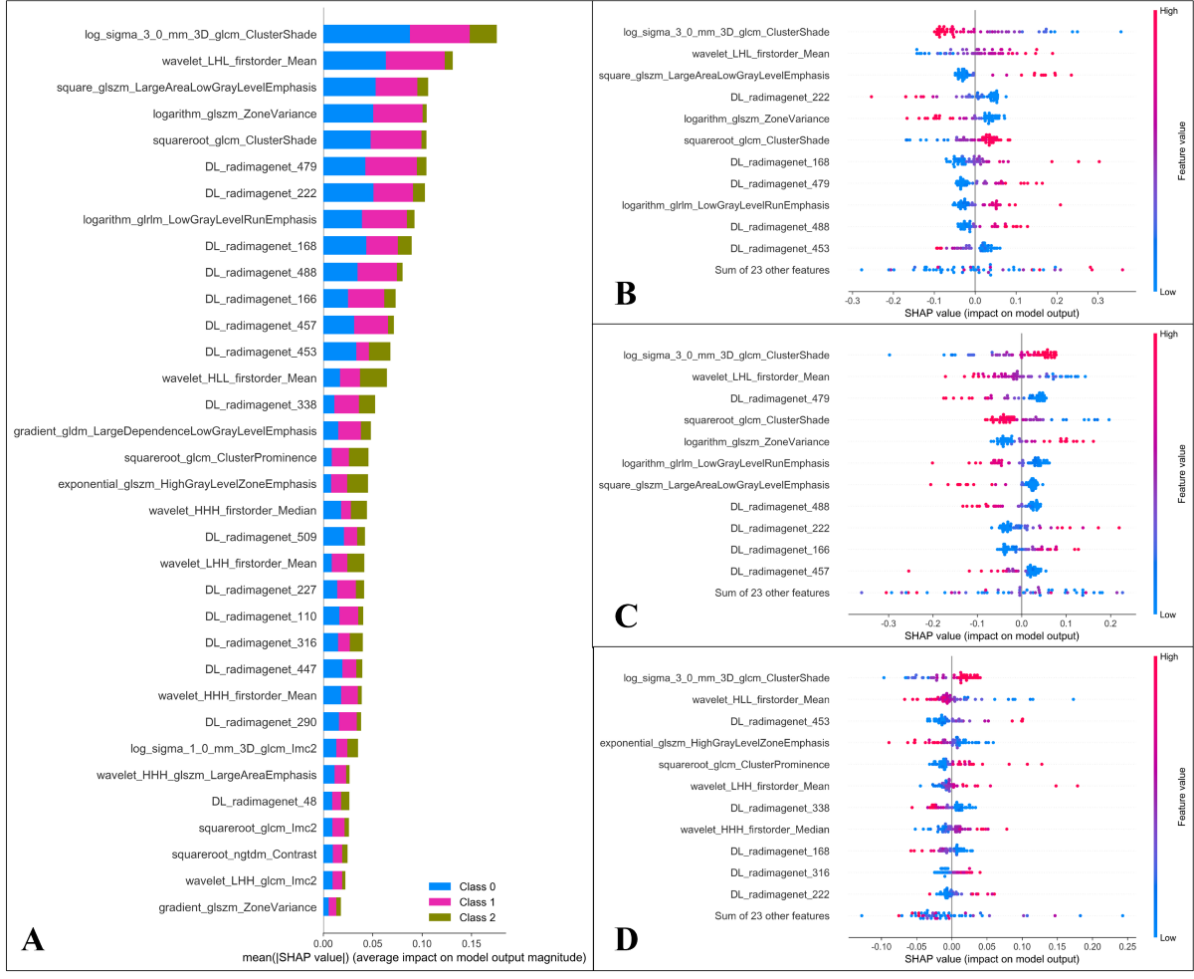
The feature contributions of the optimal fusion model are visualized as follows: The y-axis displays features arranged in descending order based on their mean absolute impact on the predictive model. The Shapley Additive Explanations (SHAP) value of a specific feature is represented by its distance from x=0, where a greater distance signifies a stronger impact—either positive or negative—on the model’s output. Each point’s color corresponds to the original value of that feature, transitioning from low (blue) to high (magenta) on the color scale. (**A**) The global feature contribution bar chart illustrates the contributions for the 3-class classification, with blue, red, and dark green bars indicating classifications 0, 1, and 2, respectively. (**B, C, D**) Beehive summary plots depict the decreasing feature contributions for predictions corresponding to classifications 0, 1, and 2, respectively.

**Figure 8.**
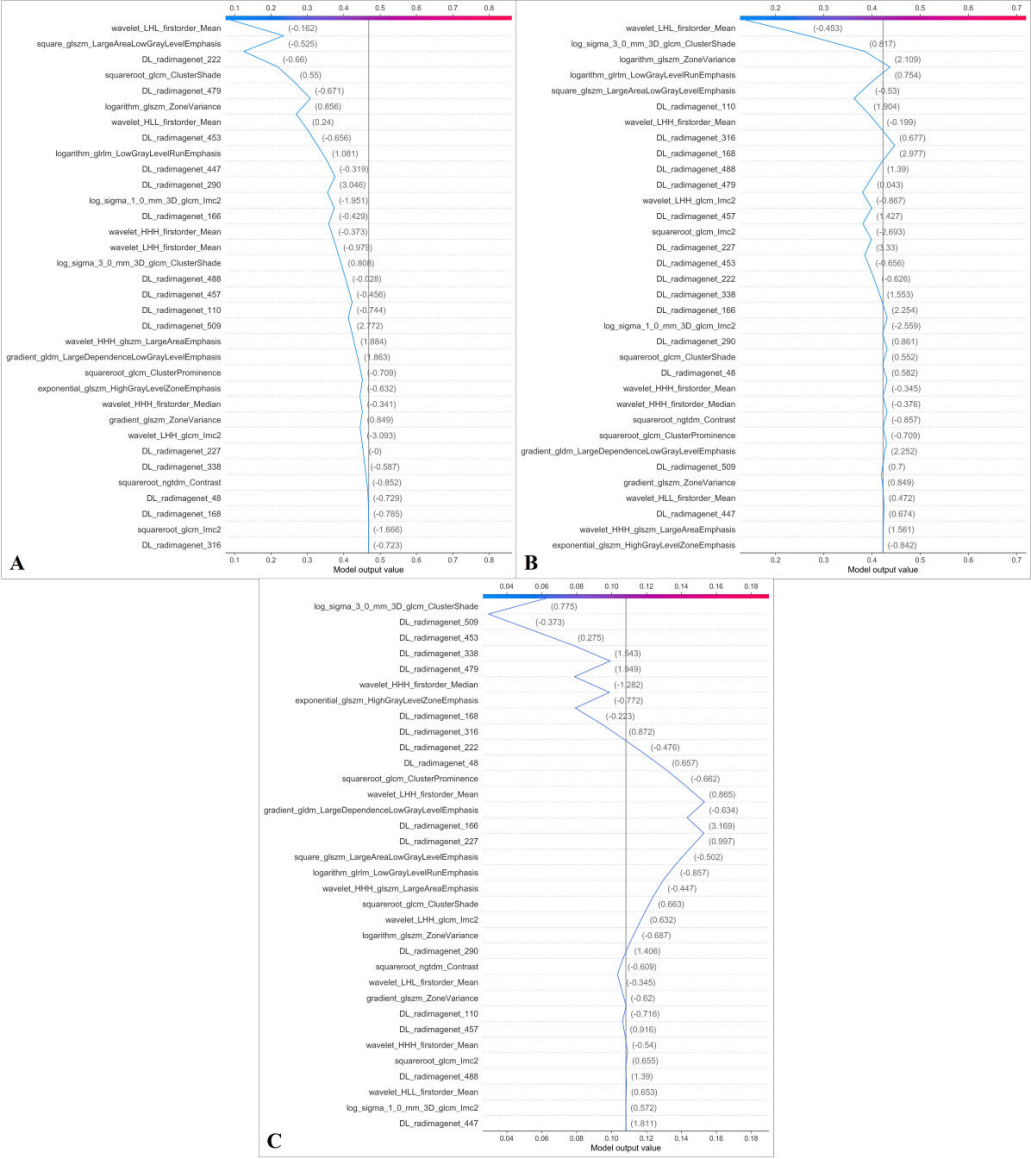
Shapley Additive Explanation (SHAP) decision plots for the 3 classifications (A: Class 0, B: Class 1, C: Class 2) are presented. The x-axis represents the model output, while the y-axis lists the feature names. The gray vertical line at the center shows the baseline value. Each line traces the prediction process, starting from the baseline value and incorporating the contributions of various features, both positive and negative, to arrive at the final model output. For classification 0, the baseline value is 0.468, with a final model output of 0.090. For classification 1, the baseline value is 0.423, with a final model output of 0.138. For classification 2, the baseline value is 0.108, with a final model output of 0.062.

The SHAP value for each feature was calculated. Figure 7 presents the global SHAP values for both the overall 3-class classification and each specific class, evaluating their impact on the model’s predictions. The highest-ranked features were cluster shade, mean, and large area low gray level emphasis. Figure 8 presents the SHAP decision plot, illustrating the prediction model’s workflow in classifying Class 0 (male, 65 years; ASTLOF 2 points), Class 1 (female, 72 years; ASTLOF 4 points), and Class 2 (female, 68 years; ASTLOF 6 points) within the prospective validation set.

## Discussion

### Principal Findings

This study developed and validated a DLR model based on CT imaging data from multiple medical centers for the classification of OVFs according to the ASTLOF system. By integrating radiomics and DTL features extracted from both RadImageNet and ImageNet datasets, the fused model—especially when using RadImageNet pretraining—demonstrated superior predictive performance across internal, external, and prospective validation cohorts. The model achieved the highest accuracy in identifying Class 2, with SHAP analysis indicating that features such as cluster shade, mean, and large area low gray level emphasis played the most significant roles in prediction. These findings highlight the model’s robustness and generalizability, supporting its potential utility in guiding clinical decision-making for OVF classification and treatment planning.

### Study Implications

Compared with obvious traumatic vertebral fractures, OVFs are an insidious condition and are often misdiagnosed. Improper treatment can affect spinal stability and balance, and in severe cases, lead to neurological dysfunction and increased risk of mortality [29]. A scientific classification of OVFs is the prerequisite for appropriate treatment. However, existing classification methods are primarily based on classification systems for early thoracolumbar fractures (which do not differentiate between traumatic and osteoporotic vertebral fractures), resulting in confusion in the treatment of OVFs [30]. These methods fail to adequately consider the characteristics of osteoporotic vertebrae, are overly complex, and lack widely accepted unified standards. Some even overly emphasize surgical treatment. A systematic classification of OVFs is crucial for assessing fracture risk, guiding treatment decisions, and forecasting patient outcomes [31]. An ideal classification system for OVFs should encompass several essential features to ensure comprehensive and practical utility. First, it should integrate imaging parameters from X-rays, CT, and MRI, enabling a thorough multiperspective assessment of the fractures. Second, it must incorporate patients’ clinical presentations, such as lower back pain and neurological symptoms, to provide a holistic understanding of the condition. Third, the system should offer treatment guidance tailored to each classification type, facilitating targeted clinical interventions. Fourth, it is essential that the system demonstrates high reliability and reproducibility, ensuring consistent application across different clinical settings. Finally, it should effectively evaluate the severity of the condition and provide prognostic insights based on classification outcomes. The ASTLOF classification system provides a thorough framework for evaluation by integrating vertebral morphology, MRI signal characteristics, BMD, and clinical symptoms. Through its scoring system, it enables clinicians to select targeted treatment plans, streamlining clinical workflows while delivering significant clinical guidance. Furthermore, existing studies have validated the system’s high consistency and reproducibility, reinforcing its effectiveness in guiding clinical treatment decisions.

Recent advancements in artificial intelligence have shifted OVF classification research toward detection, with studies showing that deep learning and radiomics methods significantly surpass traditional visual analysis approaches [32]. Most current research on OVF classification detection primarily uses single-center data split into training and validation sets for internal validation. This method is constrained by significant variability in radiomics analysis results due to differences in imaging techniques, postprocessing, reconstruction workflows, and scanning parameters across devices from various manufacturers [33]. In addition, single-center studies often lack data heterogeneity, increasing the risk of overfitting and reducing the generalizability of the findings. By contrast, multicenter studies leverage diverse imaging data, and predictive models validated using independent external datasets better account for the heterogeneity of OVFs, offering results that are more aligned with the principles of precision medicine. Our study’s strength is the use of CT imaging data from various hospitals combined with the ASTLOF classification system. A fused predictive model integrating radiomics and DTL features was developed using datasets such as RadImageNet and ImageNet. The model was thoroughly evaluated for its predictive performance in OVF classifications, offering a robust and generalizable framework for clinical application.

Studies indicate that the RadImageNet dataset notably improves DTL performance in medical applications, offering superior generalization over conventional datasets [34]. Our study’s findings confirmed that prediction models using the RadImageNet dataset surpassed those using the ImageNet dataset. In situations where sample sizes are imbalanced, the “OvR” strategy is commonly used for 3-class classification tasks [35]. In this study, the use of the “OvR” strategy in prediction models for CT images proved to be highly effective. Notably, classification 2, despite having a smaller sample size, was identified with the highest accuracy. The enhanced spatial and density resolution of CT images enables prediction models to more effectively identify radiomic and DTL features. These results highlight the potential of leveraging high-resolution imaging data and advanced datasets such as RadImageNet to achieve robust and accurate predictions, even under the challenge of imbalanced samples.

### Comparison to Prior Work

Finally, our study used SHAP values to evaluate the importance of features. SHAP values indicate the positive or negative contributions of each predictive variable to the target variable [36]. Based on game theory, SHAP is a classical post hoc explanation framework used to analyze typically incomprehensible black box models. Aggregating SHAP values across features offers a comprehensive view of each feature’s impact on the model’s predictions, clearly explaining the decision-making process. In this study, the feature with the highest contribution in CT images was cluster shade, which measures the skewness and asymmetry of the intensity distribution of grayscale in an image. It is inversely proportional to the number of asymmetric densities in the image. High skewness in the co-occurrence matrix results in lower cluster shade values, while smaller cluster shade values suggest greater homogeneity in the distribution of lesions [37]. We hypothesize that a smaller cluster shade value indicates a more homogeneous distribution of lesions within the vertebral body. While our model incorporates SHAP analysis to provide post hoc interpretability, we recognize that the deep learning component remains largely a “black box,” which may limit clinician trust and acceptance. We acknowledge the importance of further enhancing the model’s explainability—particularly in elucidating how deep features correspond to specific anatomical or pathological findings relevant to ASTLOF classification. In future work, we intend to explore and integrate advanced interpretability techniques such as attention maps, layer-wise relevance propagation, and feature visualization. These methods have the potential to provide more granular and intuitive explanations for the model’s predictions, thereby facilitating broader clinical adoption and understanding.

### Limitations

Although this study has achieved certain results, it still has some limitations. First, a key limitation of our study is the unequal distribution of cases among the ASTLOF classifications, particularly the small sample size for Class 2. This class imbalance may affect the statistical power and generalizability of the model for underrepresented classes. Moving forward, we intend to increase the sample size for each class and explore robust solutions such as synthetic oversampling, class weighting, or other augmentation strategies to enhance model performance and clinical applicability across all categories. Second, the lack of interpretability of deep learning features limits its widespread clinical adoption and trust to some extent. Moving forward, strengthening research on the interpretability of deep learning features will be crucial. This will improve model transparency, foster clinician trust, and guide clinical decision-making, thus enhancing the practical application of deep learning in medical imaging diagnosis and treatment planning. Third, our study’s dataset was exclusively sourced from Chinese hospitals, which may introduce geographic or ethnic bias, potentially limiting the generalizability of the findings to other regions and populations. Imaging protocols, equipment, and patient demographics may differ significantly across health care systems worldwide. Future work will focus on expanding our dataset with more diverse, multinational samples and performing external validation in independent international cohorts. Such steps are essential for demonstrating the model’s robustness and ensuring clinical applicability on a global scale.

### Conclusions

Compared to the fusion feature model (ImageNet), the fusion feature model based on CT images (RadImageNet) demonstrated higher predictive performance. Notably, it achieved the best performance in identifying classification 2, followed by classifications 0 and 1. This may have significant clinical value for predicting OVF classifications and guiding the formulation of treatment plans.

## Supplementary material

10.2196/75665 Multimedia Appendix 1 The computed tomography and magnetic resonance imaging acquisition parameters of the 3 centers.

10.2196/75665 Multimedia Appendix 2 A basic architecture of a convolutional neural network.

10.2196/75665 Multimedia Appendix 3 Schematic diagram of the deep convolutional neural network pretraining and fine-tuning network structure.

10.2196/75665 Multimedia Appendix 4 Feature selection was performed using the Least Absolute Shrinkage and Selection Operator (LASSO). Left: Histogram of feature importance scores based on fused features. Right: Curve of feature coefficients varying with λ, with the optimal λ value being 0.0031.

10.2196/75665 Multimedia Appendix 5 DTL_Radscore: The y-axis represents the selected fused features, while the x-axis represents the correlation coefficients.

10.2196/75665 Multimedia Appendix 6 Feature selection was performed using the Least Absolute Shrinkage and Selection Operator. Left: Histogram of feature importance scores based on fused features. Right: Curve of feature coefficients varying with λ, with the optimal λ value being 0.0295.

10.2196/75665 Multimedia Appendix 7 DTL_Radscore: The y-axis represents the selected fused features, while the x-axis represents the correlation coefficients.

## Abbreviations

ACC: accuracy
ASTLOF: Assessment System of Thoracolumbar Osteoporotic Fractures
AUC: area under the curve
BMD: bone mineral density
CT: computed tomography
DLR: deep learning radiomics
DT: decision tree
DTL: deep transfer learning
DXA: dual-energy X-ray absorptiometry
ExtraTrees: extremely randomized trees
ICC: intraclass correlation coefficients
KNN: k-nearest neighbor
LASSO: Least Absolute Shrinkage and Selection Operator
LightGBM: Light Gradient Boosting Machine
LR: logistic regression
MLP: multilayer perceptron
MRI: magnetic resonance imaging
OVFs: osteoporotic vertebral fractures
OvR: One-vs-Rest
RF: random forest
ROC: receiver operating characteristic
SEN: sensitivity
SHAP: SHapley Additive exPlanations
SPE: specificity
STIR: short tau inversion recovery
SVM: support vector machine
XGBoost: Extreme gradient boosting
