# Supplementary material for: Deep Learning Radiomics Model Based on Computed Tomography Image for Predicting the Classification of Osteoporotic Vertebral Fractures: Algorithm Development and Validation
Source: JMIR Med Inform. 2025 Aug 29;13:e75665. doi: 10.2196/75665 (PMC12396830; doi:10.2196/75665)
Supplement: Multimedia Appendix 1 [file medinform-v13-e75665-s001.docx]

**Multimedia Appendix 1**. The CT and MRI image acquisition parameters of the three centers.

|  |  | **Center I** | **Center Ⅱ** | **Center Ⅲ** |
| --- | --- | --- | --- | --- |
|  | Parameters | The Affiliated Taizhou People's Hospital of Nanjing Medical University | The Affiliated Hospital of Nanjing University of Chinese Medicine | Sir RunRun Hospital affiliated to Nanjing Medical University |
| **CT system information** | CT system | 16-slice multi-detector CT scanner (Lightspeed Ultra, GE Healthcare, USA) or 128-slice Dual source CT (Somatom Definition, Siemens Healthcare, Germany) or 64-slice spiral CT (SOMATON sensation64, Siemens Healthcare, Germany) | 16-slice multi-detector CT scanner (Lightspeed Ultra, GE Healthcare, USA) or 256-slice Dual Source CT (Somatom Force, Siemens Healthcare, Germany) or 128-slice Dual Source CT (Somatom Definition, Siemens Healthcare, Germany) | 256-slice spiral CT (Brilliance iCT, ROYAL PHILIPS, Netherlands) or 64- slice multi-detector CT (Optima CT670, GE Healthcare, USA) |
| **CT scan parameters** | Tube voltage | 120 kVp or 120 kVp with  automated tube current modulation | 120 kVp or 120 kVp with  automated tube current modulation | 120 kVp with  automated tube current modulation |
|  | Tube current | 125 – 310 mA or 185 mA with automated tube current modulation | 118 – 320 mA or 180 mA with automated tube current modulation | 180 mA with automated tube current modulation |
| **CT image information** | Image matrix | 512×512 | 512×512 | 512×512 |
|  | layer thickness and layer interval | 1 mm, 1 mm | 1 mm, 1 mm | 1 mm, 1 mm |
| **MRI system information** | MRI system | Verio 3.0T (Siemens Healthcare, Germany) or Skyra 3.0T (Siemens Healthcare, Germany) or Ingenia CX 3.0T (PHILIPS, Netherlands) | MAGNETOM Prisma 3.0T (Siemens Healthcare, Germany) or Achieva TX 3.0T (Philip, Netherlands) | Ingenia CX 3.0T (PHILIPS, Netherlands) or Magnetom Verio 3.0T (Siemens Healthcare, Germany) |
|  | Echo time (TE)  Repetition time (TR) | 60–70 ms  2,800–3,800 ms | 60–70 ms  2,800–3,800 ms | 60–70 ms  2,800–3,800 ms |
| **MRI image information** | Matrix size: | 256 × 256 or 320 × 320 | 256 × 256 or 320 × 320 | 256 × 256 or 320 × 320 |
|  | Slice thickness  Slice gap: | 3 mm  0.3 mm | 4 mm  0.3 mm | 3 mm  0.3 mm |
