# Supplementary figures and images for: Deep Learning Radiomics Model Based on Computed Tomography Image for Predicting the Classification of Osteoporotic Vertebral Fractures: Algorithm Development and Validation
Source: JMIR Med Inform. 2025 Aug 29;13:e75665. doi: 10.2196/75665 (PMC12396830; doi:10.2196/75665)

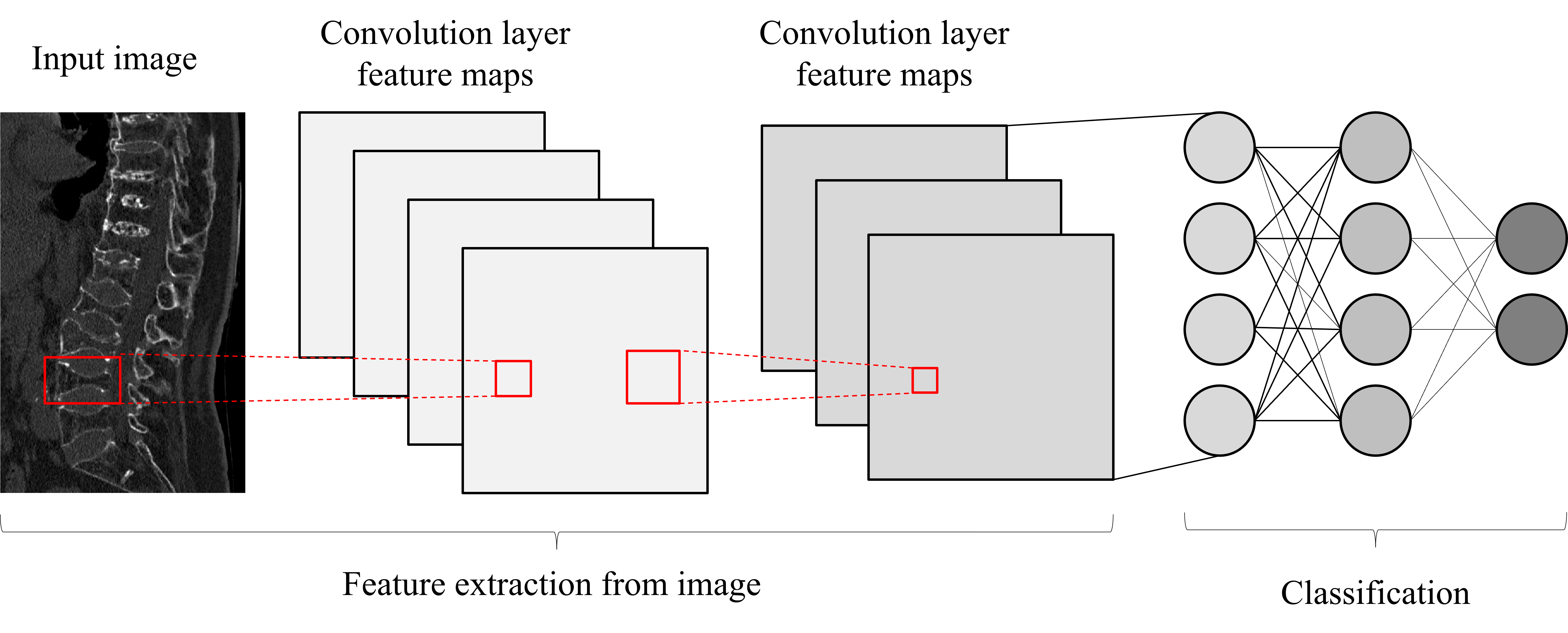

Supplement: Multimedia Appendix 2 [file medinform-v13-e75665-s002.png]

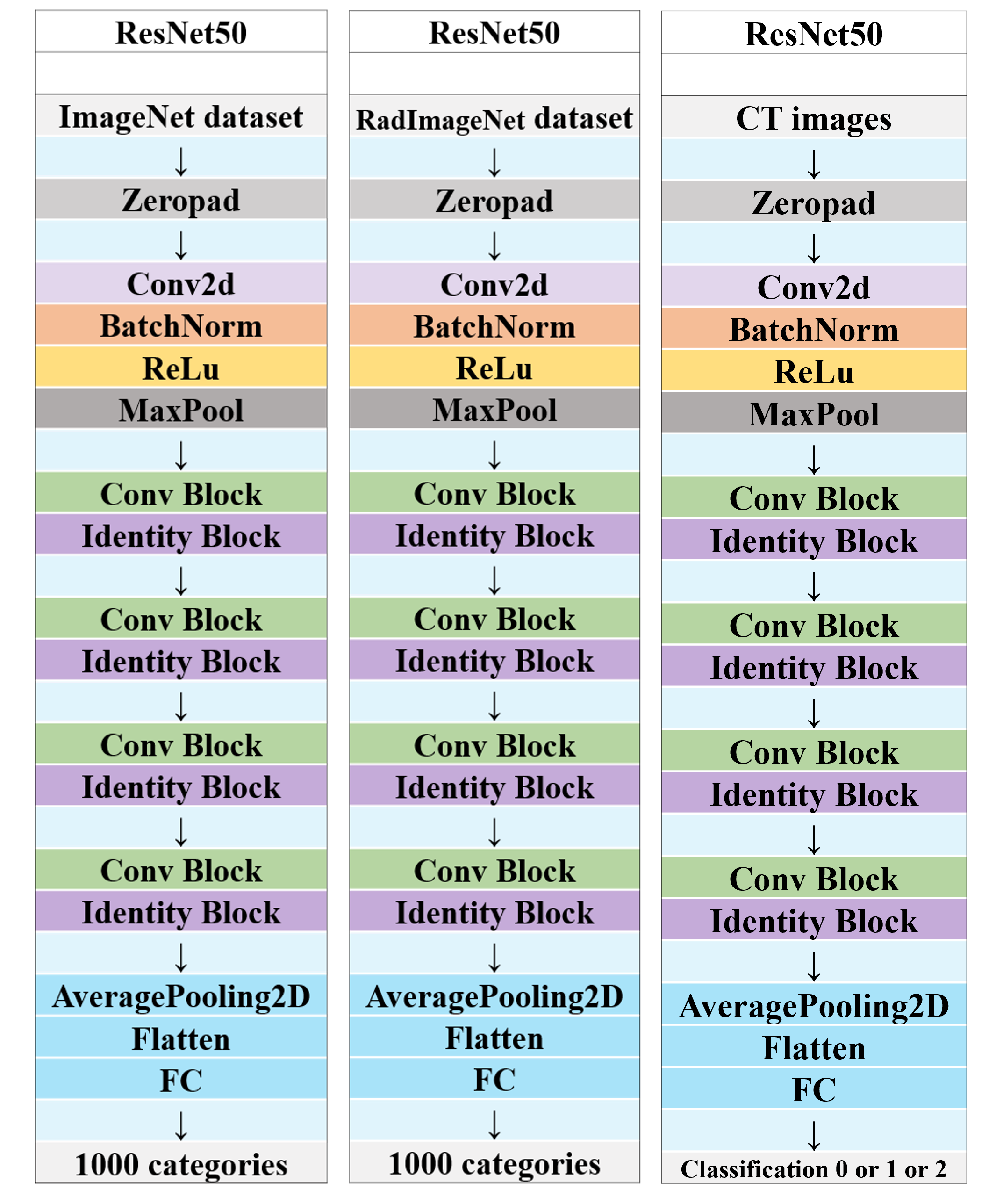

Supplement: Multimedia Appendix 3 [file medinform-v13-e75665-s003.png]

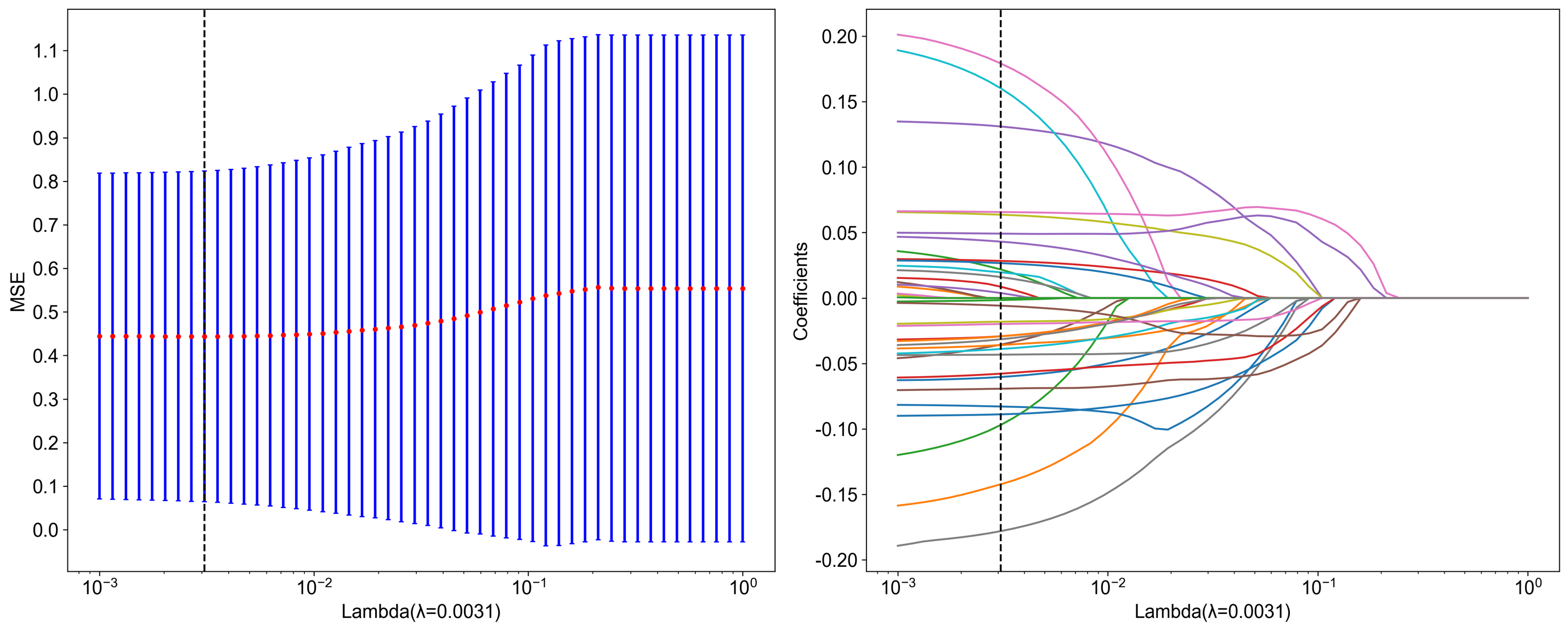

Supplement: Multimedia Appendix 4 [file medinform-v13-e75665-s004.png]

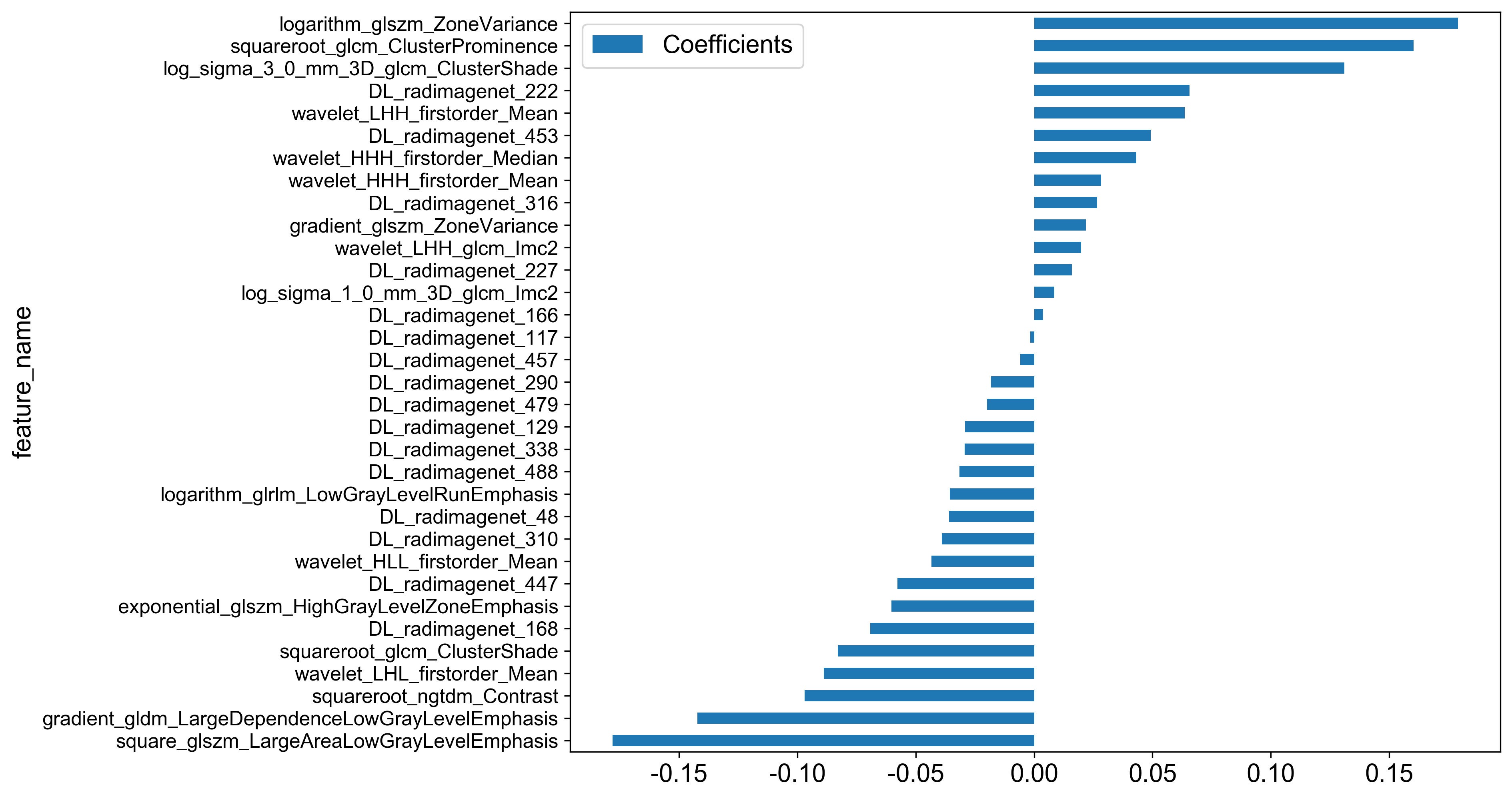

Supplement: Multimedia Appendix 5 [file medinform-v13-e75665-s005.png]

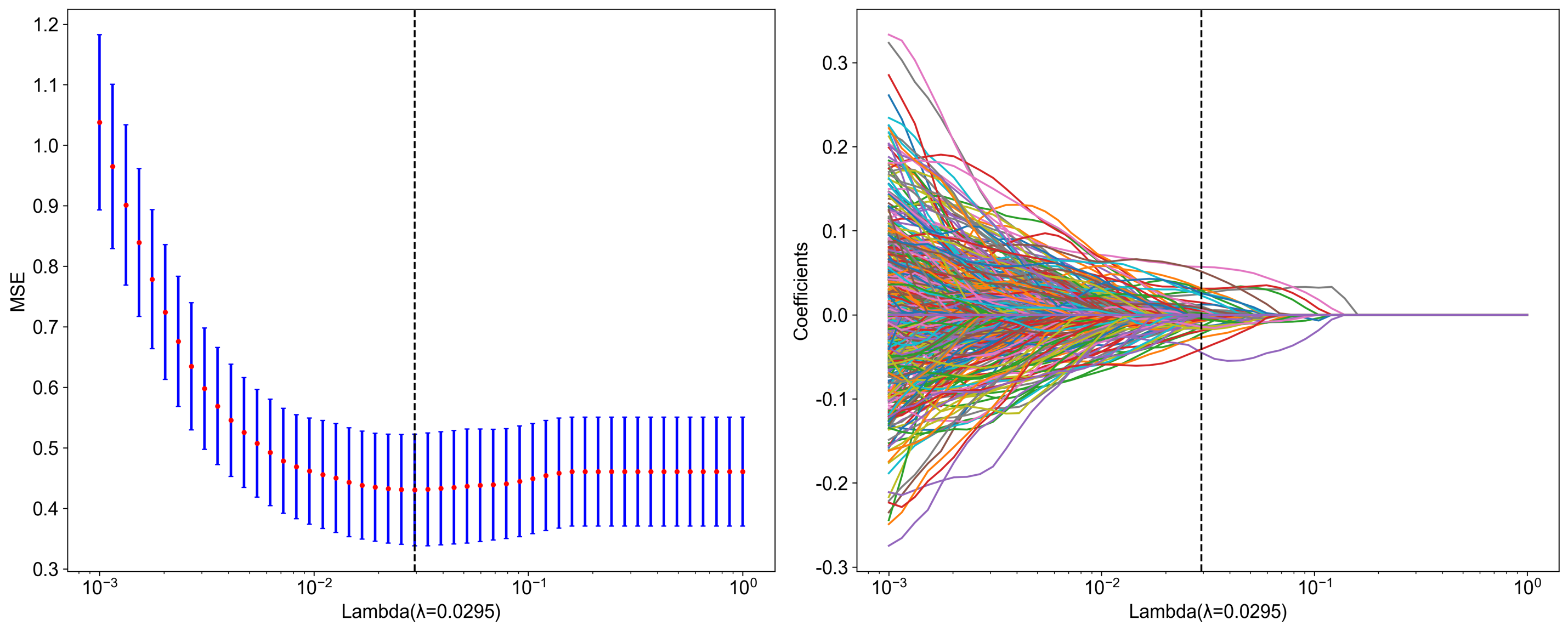

Supplement: Multimedia Appendix 6 [file medinform-v13-e75665-s006.png]

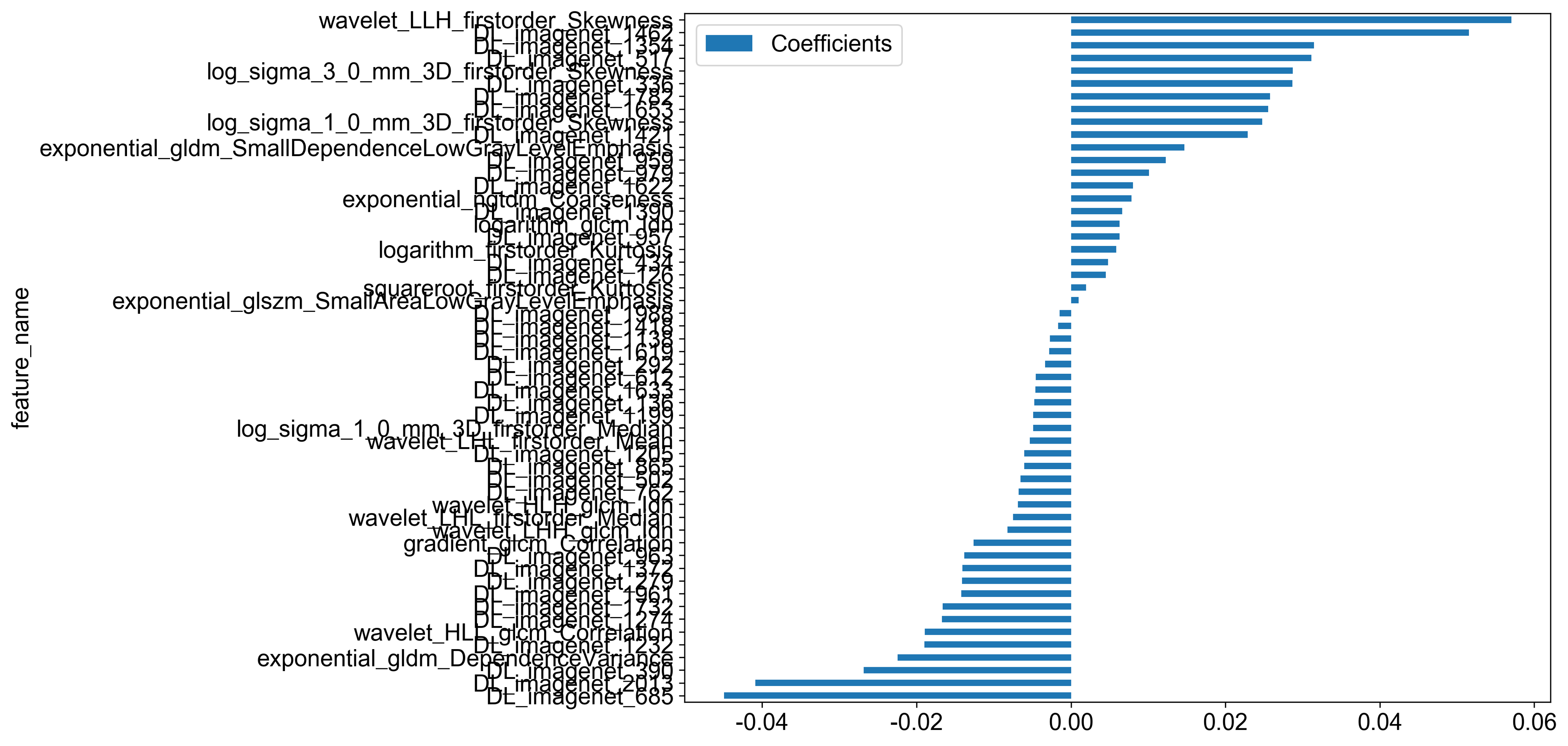

Supplement: Multimedia Appendix 7 [file medinform-v13-e75665-s007.png]
